# Community Nurses’ Perspectives on Conceptual Challenges Related to the Need for Nursing Care in Germany: A Constructivist Grounded Theory Study

**DOI:** 10.1177/15271544241228507

**Published:** 2024-01-23

**Authors:** Miriam Laepple, Margitta B. Beil-Hildebrand

**Affiliations:** 1 University of Koblenz – Faculty 1: Educational Sciences, Institute of Nursing Science, Koblenz, Rhineland–Palatinate, Germany; 2 31507 Paracelsus Medical University Salzburg – Institute of Nursing Science and Practice, Salzburg, Austria

**Keywords:** Grounded theory, ambulatory care, professional-family relations, negotiation, Germany

## Abstract

In Germany, a person's need for nursing care is assessed by evaluators according to the federal legal definition of the statutory long-term care insurance (LTCI). This definition and the associated standardized assessment tool constitute the conditions for providing nursing care in a community care setting in Germany. Furthermore, the community care setting is regulated by state law and negotiations between long-term care funds and associations of providers of nursing care. During nursing care, nurses engage in a variety of interactions with people. The extent to which the legal definition of the need for nursing care leads to challenges in these interactions is unclear. To address this knowledge gap, we conducted 22 problem-centered interviews with nurses in the community and analyzed the data using the constructivist grounded theory. The results revealed that the negotiation processes are settled within professional-family relationships and vary between the constructs of closeness and distance, advocacy and submission of responsibility, and ethos and technocracy; these are the central challenges nurses encounter in this setting. We discuss the implications and questions that arise from the findings for the nursing profession regarding its own current and future role as well as the design of nursing support in the community, to nurture more advanced nurse practitioners and community health nurses.

## Background

This article discusses the results of a constructivist grounded theory study on the challenges of nursing care from the perspective of community nurses in Germany. It addresses the interactions of nurses and sheds light on the challenges at the interface of the nursing profession and legal regulations of statutory long-term care insurance (LTCI).

Since 1995, the need for nursing care has been assessed by the Medical Review Board^1^ according to the legal definition in § 14 of the LTCI in *Sozialgesetzbuch XI* (Social Code Book XI (*SGB*), which is as follows:

…[the LTCI] covers anyone who exhibits health-related restrictions to their independence or abilities and consequently needs the help of others. It thus relates to all individuals who cannot independently compensate for or overcome physical, cognitive or psychological impairments or health-related impairments or needs. The need for long-term care must be enduring—meaning it must be expected to last at least six months. (Federal Ministry of Labour and Social Affairs, 2020, p. 114)

In 2017, the definition and its assessment tool were revised. The evaluators visited the applicants at home and assessed their need for support according to six categories: “self-care,” “coping with and independently dealing with illness- or therapy-related demands and burdens,” “organizing everyday life and social contacts,” “mobility,” “cognitive and communicative abilities,” and “behavior and psychological problems” (MDS, 2016). The six categories resulted in five care grades for affected people to receive increasing amounts of financial support for nursing benefits. The evaluators of the Medical Review Board are physicians and as well nurses, but they are independent of nursing care services in the community care setting, which means that nurses in the community are not yet in charge of assessing a person's need for nursing care.

Some indications in nursing science suggest that the tool for assessing the need for nursing care (MDS, 2016) has some weaknesses. For example, Bruehl et al. (2019, 2016) criticized the instrument for not being suitable for assigning care needs to specific care grades. These care grades define the deficit of independency or abilities: Care grade 1 addresses minor impairments, Care grade 2 means a significant impairment, going up to Care grade 5 which denotes most severe impairment of independency or abilities with special requirements for nursing care. In inpatient long-term care, facility managers have observed that people in need of nursing care are now classified as having a lower care grade, compared to the previous care level system (Isfort et al., 2018). The level of approved care grades of residents in facilities is a decisive criterion for the number and refinancing of nursing personnel (Isfort et al., 2018; Rothgang et al., 2017). Furthermore, the assessment lacks an empirical basis, thus the instrument cannot contribute to the further development of nursing science and theory (Bruehl & Planer, 2019; Bruehl et al., 2016).

In the community care setting in Germany, people in need of nursing care remain in their familiar home environment, often on their own (Dahlin-Ivanoff et al., 2007). With regard to the nursing relationship, it can be assumed that there are many different interactions between nurses and people in need of care and their relatives, which also affect the need for nursing care. In this context and from the perspective of nursing science, some important questions arise: What effect does a legal definition of the need for nursing care and the associated assessment procedure have on nursing interactions with people in need of care? Do nurses in the community possibly have their own approaches to address the need for nursing care? A systematic international literature review revealed some findings regarding the extent to which the need for nursing care leads to challenges for the actors involved; these relate to interface work in the community care setting (Gágyor et al., 2019), informational aspects for organizing care (Drossel et al., 2020; Roehnsch & Haemel, 2021), a concept analysis of the term “care dependency” (Boggatz et al., 2007), and the cooperation between nurses and family caregivers (Buescher et al., 2011). These studies refer to the legal definition of the need for nursing care before its reform in 2017; to our knowledge, no study has examined its conception since 2017, regarding the challenges for interactions within care. Furthermore, Laepple (2022) pointed to the lack of findings on nursing assessments of the need for nursing care in the German community care setting. To address this research gap, we developed the following research question:

To what extent does the legal definition of the need for nursing care present a challenge for nurses in German community nursing care?

The main research question was divided into three sub-questions: First, how do nurses in the community assess the need for nursing care; second, to what extent is the need for nursing care significant for nurses in the community; and third, what role does the self-assessment of nurses in the community play in the assessment of the current concept of the need for nursing care?

This research aimed to explore the construct of the need for nursing care at an interpretative level with regard to nursing science, demonstrate how nurses approach the need for nursing care, and develop a theory about the challenges of the legal definition of the need for nursing care based on nursing interactions.

## Design, Methodology, and Methods

This research^2^ was conducted by the corresponding author as part of a PhD thesis. We recruited nurse participants through written invitations for interviews. The invitation was distributed through a German professional association for nursing professionals and through social media. During this period, interested nurses contacted the researcher and introduced themselves by email with information about their professional experience in community care settings, educational qualifications, and specific roles in their nursing work. Inclusion criteria were completed vocational training in nursing and currently working in community care setting. Participants consisted of 15 female and 7 male German nurses aged 29–64 years. There were eight nurse directors, three nurses with further specific training (e.g., palliative care) and five nurses studying pedagogy or management of nursing. Exclusion criteria were insufficient language skills in German and ongoing vocational training.

Educated community nurses in 5 of 16 states in Germany participated in this study. This led to a rich amount of qualitative data, because the framework of the provision of nursing care varies across the states. All the participants provided written informed consent prior to engaging in this study.

### Design

We employed an exploratory interview study design and performed data analysis using the constructivist grounded theory approach (Charmaz, 2014; Corbin & Strauss, 2008). Data collection initially began with face-to-face interviews; however, following the outbreak of COVID-19 and the resulting contact restrictions, telephone interviews were conducted. Related requirements, such as the verbalization of non-verbal actions (e.g., turning on and off the audio recording), were incorporated into conversations with nurses in the community (Opdenakker, 2006, pp. 4–5; Reichertz, 2020; Scheytt, 2020).

### Methodology

Owing to its open and explorative nature, this research was substantiated using the interpretative paradigm (Keller, 2012; Wilson, 1981). Based on this foundation, the focus on interaction and meaning has resulted in a theoretical perspective of symbolic interactionism (Blumer, 1969). This focus was complemented by the choice of the constructivist grounded theory methodology (Charmaz, 2014). The constructivist aspect addresses the prior knowledge and subjectivity of the researcher, and requires the reflexivity of the researcher's influencing assumptions and experiences, which are consciously and unconsciously incorporated into the research process. Methodologically, in the current study, this reflexive attitude was related to openness in conversations with nurses, and asking questions when nurses assumed aspects of community care to be known so that their own assessment of meaning or underlying attitudes or values could be made explicit. Pragmatically, the prior knowledge and subjective imprints of the researcher were recorded in reflexive memos and a research diary, and incorporated into the data analysis process with the help of interpretation groups in order to discuss researcher bias and consider different interpretations and category properties.

## Methods

Using an open, in-depth interview method with semi-structured interview questions, 22 problem-centered interviews (Witzel, 2000) were conducted with community nurses (AltPflG, 2000; KrPflG, 2003). The interviews lasted between 26 and 80 min and were transcribed verbatim using content and semantic transcription rules, according to Dresing and Pehl (2018).

Constructivist grounded theory, as a method for generating data-based constructs, uses several coding procedures and theoretical sampling. This leads to an iterative process in which data collection and analysis occur simultaneously (Charmaz, 2014; Glaser & Strauss, 2010). Furthermore, we used the techniques of memo-writing and constant comparison, which helped us develop concepts, categories, and sub-categories (Charmaz, 2014; Corbin & Strauss, 2008).

Theoretical sampling started with four nurses from the researcher's professional circle of acquaintances who worked in different nursing care services in the community. Interview questions in this initial stage arose from the findings of the literature review. These four interviews gave a first impression about the possible concepts of interest. Thereafter, theoretical sampling was based on the nurses’ introduction sent within their outreach to the researcher via email. The developed themes guided the researcher and this led toward new data and questions by referring to the characteristics of the nurses (e.g., sex; age; length of professional experience in the community care setting; rural or urban working area; nursing care service type: profit, non-profit, or religious organization). Interview questions were developed in terms of content to address the concepts (Charmaz, 2014) and theoretical sampling was carried out throughout the process of initial and focused coding. To structure the concepts and categories, a paradigmatic model, according to Corbin and Strauss (2008), was included. The comparative and contrasting data resulted in categories of different hierarchies that could be defined during the work process. We noted saturation in theoretical sampling after 22 interviews, when no new information could be obtained.

We used the criteria for qualitative research according to Struebing et al. (2018) to guide and evaluate our study methodology. These criteria refer to *the appropriateness* of the methodology and methods for the research questions, *empirical and theoretical saturation* to gather rich and thick data that can be theoretically abstracted, *textual performance* that involves logical and comprehensible writing and clarifying the researchers’ different roles, and *originality* of the results. Thus, they should be embedded in and connected to practical and scientific nursing. We also referred to the SRQR guidelines (O’Brien et al., 2014) in this study and the manuscript conforms to the ICMJE Recommendations.

## Results

Our data analysis revealed the following categories: “care and society,” “soul life of the *pflegehaus*,”^3^ “expertise of the nurses,” and the core category “induced polarization of expertise.”

**Figure 1.**
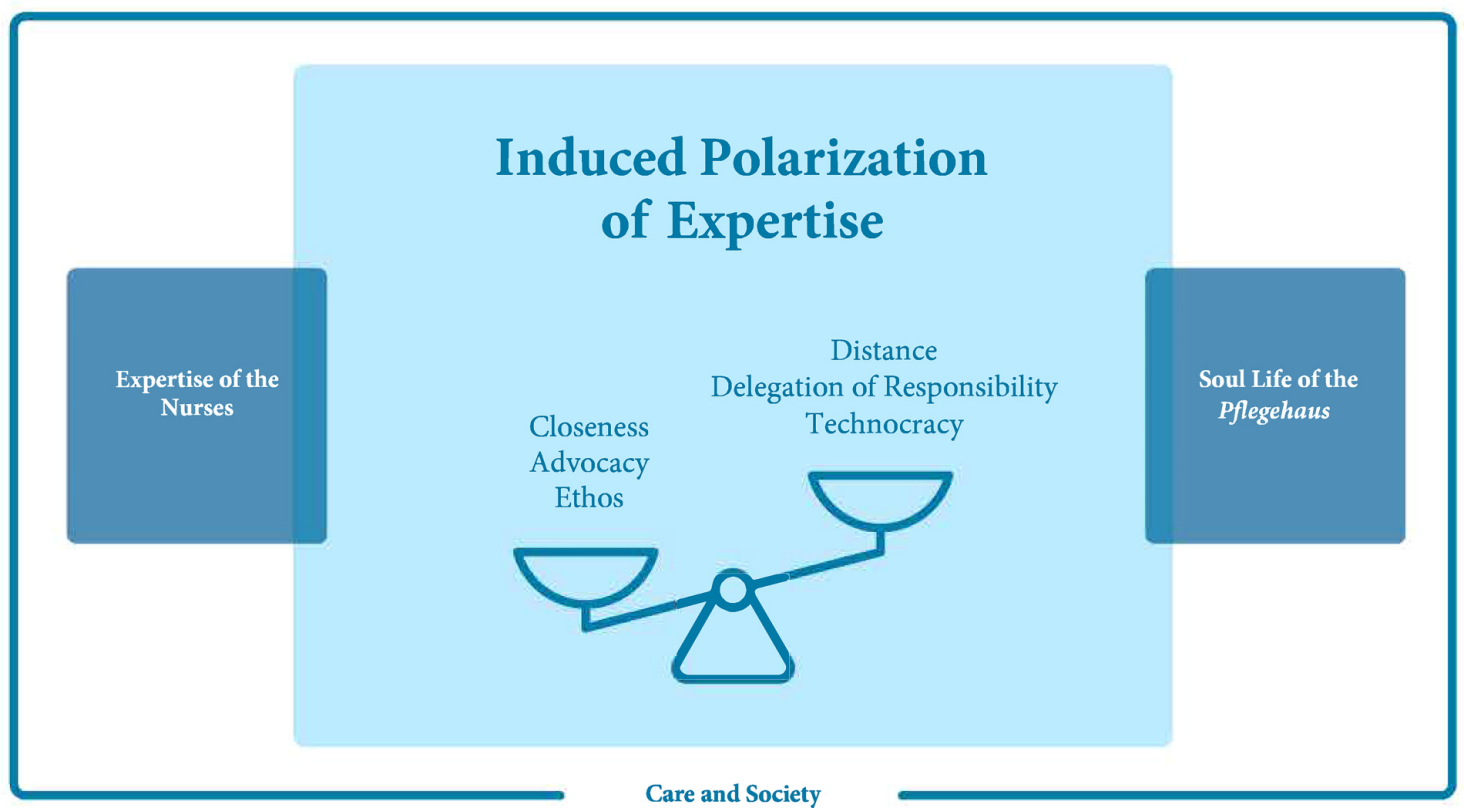
Challenges of the Concept of need for Nursing Care; © Miriam Laepple 2022.

Figure 1 presents an overview of the categories and the core category, and how they are connected to each other. “Care and society” addresses the social and structural conditions under which ambulatory care takes place in Germany. The socioeconomic status (SES) of the *pflegehaus* plays a decisive role in access to care services, for example, the possibility of applying for LTCI benefits, as well as financial, cultural, and educational resources for organizing and financing care. Family households with higher SES have less difficulty obtaining relevant information and making applications. Their working relatives are better able to organize their working hours flexibly, and there is more financial scope to finance supplementary care services privately. “Care and society” elements also affect how professional care services in the community offer these services. They are not provided as single services but as service modules. Several modules have been combined to address the greater need for care. Owing to the time values assigned to the modules and the economically justified use of synergistic effects by the nursing care service, there is time pressure on nursing personnel. This category also included aspects of the presentation of nursing topics in German media. The mostly negative image of inpatient long-term care generates the desire for persons in need of nursing care to stay at home for as long as possible. This does not change, even when home care conditions are poor.

The category “soul life of the *pflegehaus*” reveals the insights that nurses have regarding intimate and private aspects of the families. A major topic is financial resources and how this leads to shameful situations in nursing interactions, in that people in need of nursing care try to avoid using some care services. The nurses spoke about strategies that people used to try to conceal evident care needs, despite the fact that they felt lonely and often lacked skills in self-care and organized daily living. While the nurses acted as guests, their insight into situations in the *pflegehaus* made them feel responsible for providing supplementary domestic or nursing care services, so that people in need of care did not feel undersupplied or unsupported at home. The experience of declining abilities and competencies leads to people in need of care limiting their contact with society. This occurs gradually; for example, some people in need of care can no longer leave the house and are dependent on telephone calls. However, if they hear very little on the telephone despite hearing aids, the number of calls will also decrease, making people increasingly lonely.

The category “expertise of the nurses” describes how nurses assess the need for nursing care based on their professional backgrounds. Compared to the evaluation by the Medical Review Board, nurses reported prioritizing interactions with the person in need of care to generate a relationship of trust. Only through this relationship of trust can they obtain further information on hidden or shameful problems related to nursing care. Additionally, the professionally assessed need for nursing care fits the individual and domestic situations of the *pflegehaus*. Nurses used various criteria to assess the need for nursing care. Based on nursing theories, nurses are alert to the status of the person's appearance, mobility, cognition, language, and communication. They also used individual strategies to gather information. For example, they tried to understand the relationships and dynamics within the *pflegehaus*, or checked secretly the cleanliness of the toilets to detect pretended independence as they consider dirty toilets to be a hint for unmet self-care. Another strategy was the question if people in need of nursing care were playing musical instruments to gain an impression about related and required competencies, e.g., lung volume or mobility of fingers. From these sources, they drew conclusions about obvious and hidden nursing problems and resources and got in touch on a personal level. Thus, the need for nursing care is constantly evaluated by observing the situation, and different care services are introduced into the service portfolio based on trust.

The core category, “induced polarization of expertise,” describes the extent to which expertise is polarized based on the three previous categories and how it develops in the interaction process. For this purpose, we used the image of three scales, described as challenges to the legal definition of the need for nursing care from the perspective of nurses.

The first scale, closeness and distance, describes the creation of closeness as a basic condition for the relationship of trust between the *pflegehaus* and nurses. Through closeness, relevant information about the need for nursing care becomes accessible and care can be adapted to the conditions of the *pflegehaus*. However, this also requires professional distancing on the part of nurses to remain capable of acting and reacting. Problematic interactions occur especially when people with mental illnesses and feelings of loneliness are cared for by nurses maintaining little distance from them situations of mutual assault and violence can occur.

The second scale is the interaction between advocacy and the delegation of responsibility. Advocacy describes the nurse's commitment to the needs of the *pflegehaus*, often beyond finishing work. Commitment addresses not only nursing aspects but also all actions to continue living in one's own home. This scale also concerns the coordination and social functions of nursing care, which are not included in the service modules of professional nursing care services. Acting according to advocacy covers communication with general practitioners, family doctors, relatives, or the Medical Review Board to influence the care grade. In contact with relatives, it becomes clear that there is often a lack of clarity about the distribution of tasks and responsibilities within the cooperation between relatives and nurses, which can lead to undesirable events, misunderstandings, and mistrust. Advocacy depends on the interaction in the *pflegehaus* and the regulation of closeness and distance. If there is a problematic interaction on this scale, it is possible that the nurse may leave the *pflegehaus* because of poor communication.

Finally, the third scale describes nursing attitudes that oscillate between ethos and technocracy. Ethos describes the extent to which nursing interactions are guided by respect, interest, and perception of each other in equal terms, as well as respect for human dignity. These interactions are closely linked to the advocacy scale. They include an additional intrinsic value foundation so that nurses can take a firm position to stand for the concerns of the *pflegehaus*. An example of this is the nurses’ protection of the *pflegehaus* from nursing colleagues or the larger nursing care service if the business structures mainly pursue economic incentives and the needs of the *pflegehaus* are insufficiently addressed. Nurses with such an ethos-based attitude recommend their own clients to other nursing care services or try to protect them from colleagues by openly communicating deficiencies in nursing care to the management. In contrast, technocracy states that nurses interact with a technocratic attitude in the *pflegehaus*. This is characterized by processing the agreed-upon service modules without further commitment. The implicit needs of the *pflegehaus* are intentionally overlooked or not passed on, with contractually obliged tasks executed rather mechanically and without adequate attention and care given to the hidden or shameful issues; thus, a technocratic attitude is closely connected to a delegation of responsibility. The organizational structures and decisions of nursing care service managers contribute to the development of ethos and technocratic attitudes among nurses. If nurses experience unclear communication and responsibilities in the management of nursing care services, a technocratic attitude manifests through non-commitment, lack of communication, and lack of transparency regarding responsibilities and structures.

The three scales are mutually dependent and influenced by the categories “care and society,” “soul life of the *pflegehaus*,” and “expertise of the nurses.” The practices of the management of nursing care services; time pressure; the structural, social, and media conditions under which community care takes place; nurses’ educational qualifications; and the hidden and intimate aspects of family households are constitutive features of how nursing care interactions align with and are polarized by the three scales in the *pflegehaus*.

## Discussion

Based on the constructivist grounded theory, this study provides insights into nursing interactions in the *pflegehaus* community care setting in Germany. This setting is regulated on the one hand by federal law in the Social Code Book XI, stipulating the normative concept of being in need of nursing care (§ 14 SGB XI, 1994). The statutory LTCI only partially covers the costs of nursing care. On the other hand, it is framed at the state level by negotiations on prices and setting time values for delivered nursing care. These negotiations take place between long-term care funds and associations among providers of nursing care services (Bluemel et al., 2020, pp. 176–179). Currently, the normative definition of being in need of nursing care leads to a single assessment of approximately 30 − 60 min by a representative (stranger) from the Medical Review Board. Without a basis of trust, people in need of nursing care will not talk about their real, often hidden, and shameful health care problems to this stranger. Nurses gather this information during interactions. However, community nurses are not in charge of shaping community nursing on a professional or legal basis, which is determined by issues of pricing and efficiency.

Our results on the challenges of the need for nursing care showed that nurses could contribute significantly to the assessment of the need for nursing care. They are trustworthy, gaining confidence and access to people in need of nursing care easily, and through their insight into the *pflegehaus*, they plan tasks for adapting nursing care to individual and familiar circumstances. In the category “care and society,” it became clear that the SES of the *pflegehaus* played an important role in accessing (nursing) care services and further information. That is, community care settings require additional uncomplicated and easy-going structures to inform people with low SES about their possibilities before a nursing care arrangement is implemented.

If nurses in the community are responsible for assessing the need for nursing care on a legal basis, barriers at interfaces and bureaucracies for affected people could be overcome. Nursing care can be more suitable, sustainable, flexible, and affordable for affected people because it can be adapted to their needs. The steady presence of nurses in the *pflegehaus* enables a dynamic and flexible evaluation of the condition and health status of people in need of nursing care. Consequently, the costs of deterioration, decompensation, and inpatient hospital stay could be avoided. Furthermore, in Germany, nursing as a profession could become more attractive because the definition of nursing-reserved tasks, such as the assessment of the need for nursing care, brings more responsibility, scope of action, and professional relevance to social and financial insurance issues.

Certainly, there should be a nursing association that manages nursing assessments on the need for nursing care to support and relieve nurses in community care services. This association should also scientifically develop and evaluate nursing and SES assessments so that the distribution of long-term care funds is based on a transparent, professional, and scientifically developed foundation. Additionally, access to people with low SES should be facilitated and an assessment should be designed to record this need of information and organization, to be able to offer suitable care services.

This research shows the hidden scope of nursing interactions; the scales of advocacy and delegation of responsibility, as well as ethos and technocracy, convey in their contents a nursing care beyond the mere processing of contractually stipulated service modules. This affects relationship processes with people in need of nursing care, their relatives, and third parties (e.g., general practitioners, local providers, and housekeeping) who are involved in the care in the background. Consequently, the care provided is oriented toward people, their environment, their conditions, and their wishes and needs, and generates deep trust within the interpersonal relationship. However, this is not a regular process but depends on the individual nurses and the scope for structuring their work, as it is organized by their employers.

In addition to the discussion of the structural setting issues presented above, the findings also discuss the role of nurses in community care settings within the nursing profession. The nursing visits stipulated in § 37(3) SGB XI serve as an example, during which nurses check the extent to which recipients of financial support from the LTCI use this money to finance and organize care. If a person in need of nursing care is found to be underprovided during this visit, nurses are obliged to provide counselling and report the situation to the LTCI. In the event of sanctions, financial support would no longer be redeemed, but a nursing care service would provide in-kind benefits within the scope of the care grade. The challenge for nurses can be found in the following questions: To whom can nurses commit themselves? What effects do these control functions have on nursing interaction and the relationship of trust in the *pflegehaus*? This dichotomy is neither reflected nor evaluated by nurses. Again, this issue requires an association of nurses in the community to moderate and discuss these implications on a professional and self-governmental basis.

Within the nursing assessment of the need for nursing care, it is also striking that nurses do not orient themselves to theoretical models, such as nursing theories, but rather act in a performance-related manner. This results from a contractually defined portfolio of services and is implicitly reflected in the advocacy and nursing ethos of nurses. However, interactions going beyond the service modules are not the result of a negotiation process between the nurse and the *pflegehaus* but result from the personal estimation and judgment of the nurse. Furthermore, nurses rarely consider abstract topics such as spirituality and gender aspects or reflect on their own role and influence on interactions in the *pflegehaus*.

Understanding the consequences of nursing interactions is essential for people in need of nursing care to remain in their homes. This concerns both the positive consequences of an assumed advocacy and a nursing attitude based on ethics, as well as the negative consequences, especially relating to responsibility at the interfaces of care; it is already known from other publications that misunderstandings, mistrust, and errors repeatedly occur between nurses, caring relatives, and physicians (Buescher et al., 2011; Gágyor et al., 2019). In addition to the structural changes mentioned above, nurses in the community must take care of transparent responsibilities regarding the organization and arrangement of nursing and family care. These concerns include the organization of aids, prescriptions, medications, and appointments with therapeutic professionals. A clear agreement on these accompanying tasks enables nurses to perform them in a professional manner and helps save costs in community care settings.

This study has the following strengths and limitations. It is centrally located in a community care setting, and can be transferred to other settings in a theoretically abstract manner through further empirical and theoretical considerations. It can be assumed that other challenges are associated with the need for nursing care in both acute and inpatient long-term care. Our results are based on interview data with nurses in a community in Germany. More perspectives could be explored if people in need of nursing care and their relatives contribute to further analyses of the need for nursing care. Finally, the corresponding author had worked for several years as a nurse in a community care setting; thus, there was a corresponding imprint and prior knowledge on the constructivist character of the research. However, in the research process, this contributed to good access to the field of study and promoted trust between the nurses in the community and the researcher in their conversations. This made it possible to bring up and discuss taboo and challenging topics.

A starting point for further research could be nursing assessment of the need for nursing care. The developed criteria could be tested in an operationalized and questionnaire-based manner in the nursing profession. It is possible that hierarchies or weightings result from this complementary approach; thus, the empirical results conceptually serve for the development of an assessment instrument. The extent to which this instrument is suitable to statistically explain the need for nursing care should also be examined. With this approach, discussions on the distribution of LTCI funds through self-governance associations of nurses should be reinitiated.

## Conclusions

Our results clarified the challenges of the concept of the need for nursing care from the perspective of nurses in German community care settings. Whether closeness and distance, advocacy, delegation of responsibility, ethos, and technocracy can be shaped by nurses is influenced by the structural, media, and financial conditions under which nursing care interactions take place. The normative assessment procedure based on Social Code Book XI has weaknesses in tailoring suitable, sustainable, and affordable nursing care services. Further, people with a low SES require additional support to gather information and organize care arrangements. Nurses with a social advocacy role in the community care setting could be responsible for ensuring that affected people receive timely and low-threshold advice on organizing care arrangements. To achieve this, it is crucial to develop community-based health structures with a new distribution of tasks within communities. Interaction-based nursing assessments of the need for nursing care must be discussed and developed by German nursing associations and nursing science. On the one hand, this would serve the nursing profession as a scientific and professional approach to assess important phenomena in the field. On the other hand, this should be introduced in German health care only if it is economic, simple, comprehensive, and supported by the state and federal governments. Prior to this, professional nursing associations and nursing politics were required to define the scope of nursing interactions with regard to responsibilities under statutory LTCI. This decision-making power requires, among other things, changes in the self-governing of nurses as well as qualitative, interprofessional, legal, and financial approaches and changes.

## Notes

1. As a state corporation under public law, the Medical Review Board has the legal mandate to support the statutory health insurance and the statutory long-term care insurance in medical and nursing care-related matters. It is supervised by the Medical Review Board of the Statutory Health Insurance Funds (MDS).

2. The Institutional Review Board of the German Society of Nursing Science (DGP) approved this research on November 4, 2019 (approval number: 19–008).

3. The term “*pflegehaus*” means the family household with all the people living or present there who are involved in the care and support of the person in need of nursing care. We use the term here to consider all actors of the household and both their evident and hidden interactions. We derived the term from our interviews with the nurses in the community.
